# Seed Bank Community under Different-Intensity Agrophytocenoses on Hilly Terrain in Lithuania

**DOI:** 10.3390/plants12051084

**Published:** 2023-03-01

**Authors:** Regina Skuodienė, Vilija Matyžiūtė, Jūratė Aleinikovienė, Birutė Frercks, Regina Repšienė

**Affiliations:** 1 Lithuanian Research Centre for Agriculture and Forestry Vezaiciai Branch, Gargzdu Str. 29, Klaipeda District, LT-96216 Vezaiciai, Lithuania; 2 Faculty of Agronomy, Vytautas Magnus University Agriculture Academy, Studentų Str. 11, Kaunas District, LT-53361 Akademija, Lithuania; 3 Lithuanian Research Centre for Agriculture and Forestry, Institute of Horticulture, Kaunas Str. 30, Kaunas District, LT-54333 Babtai, Lithuania

**Keywords:** seed bank size, species number, seed surface, agrophytocenoses, hilly relief, microbial biomass carbon

## Abstract

On the summit of a hill with a lack of humidity, and in usually stronger eroded midslope parts, crops thin out. Changing ecological conditions change the soil seed bank as well. The aim of this study was to examine changes in the seed bank size and number of species and the influence of seed surface characteristics on their spread in different-intensity agrophytocenoses under hilly relief conditions. This study included different parts of the hill (summit, midslope and footslope) in Lithuania. The southern exposition slope’s soil was slightly eroded Eutric Retisol (loamic). In spring and autumn, the seed bank was investigated at depths of 0–5 and 5–15 cm. Irrespective of the season, in the soil of permanent grassland, the seed number was 6.8 and 3.4 times smaller compared to those of cereal–grass crop rotation and crop rotation with black fallow. The highest number of seed species was determined in the footslope of the hill. Seeds with rough surfaces dominated on all parts of the hill, but the highest amount (on the average 69.6%) was determined on the summit of the hill. In autumn, a strong correlation was found between the total seed number and soil microbial carbon biomass (r = 0.841–0.922).

## 1. Introduction

The soil seed bank is the resting place of seeds and an important component of the life cycle of plants [1]. Seed banks, which contain seeds of multiple generations, are a potential source of diversity and contribute to the dynamics and persistence of the weed community [2]. Depending on the pattern of the soil seed bank, the understanding of plant succession and weed survival facilities are becoming more significant for agricultural soil [3].

Seeds are spread in the soil profile both horizontally and vertically [4]. The density of seeds in the soil is variable both in space and time [5]. Seed distribution in the soil profile depends on seed mass [6] and shape [7]. Seed mass and shape were found to be closely linked to the living conditions of plants [8]. Small spherical seeds, which penetrate the soil more easily, often display higher persistence [6,9]. Susceptibility to seed removal varies highly between species and is mainly related to seed traits, including seed size, seed shape, the presence of appendages and the ability of a seed to secrete mucilage [4]. Seeds on or in the soil may also be moved horizontally to new locations by different biotic (animals) or abiotic (often wind, runoff and gravity) agents [4].

Changes in management practices influence the distribution of seeds in the soil [10,11,12,13]. Tillage mixes seeds into the soil independent of their size and shape [8] and changes the germination environment of seeds by modifying soil cover and altering soil temperature and moisture patterns [3].

Grass vegetation is a physical and biological barrier for water runoff from a slope’s surface [14]. Rainfall, slope and soil characteristics influence seed transport [4]. In areas with high erosion risk, it is very important to form agrophytocenoses, which can be productive on hilly terrain and can protect the soil from erosion effectively [14]. Cereal–grass crop rotation (with temporary grasslands) and perennial grasslands form a permanent soil cover and improve soil conservation on slopes [15]. In our study, permanent grassland constantly provided anti-erosion protection for the slope’s soil (for over 25 years). The seed bank of permanent grassland was compared with seed banks of pre-erosion cereal–grass crop rotation (50% of perennial grasses) and crop rotation with black fallow (only 30% of perennial grasses).

There is research on seed persistence in the soil [16], dormancy [17,18], germination and emergence [19] but rarely with the attempt of establishing generic relationships between seed morphological characteristics and the parameters of hilly relief [4].

The aim of this study was to examine (1) changes in the seed bank size and number of species and (2) the influence of the seed surface characteristics on their spread in different-intensity agrophytocenoses under hilly relief conditions.

## 2. Materials and Methods

### 2.1. Site and Soil Description and Experimental Design

The experiment was carried out at the Vėžaičiai Branch of the Lithuanian Research Centre for Agriculture and Forestry, on the midslope soil of Žemaičiai Highland covered by different anti-erosion agrophytocenoses in Kaltinėnai (lat. 55°57′ N, long. 22°48′ E, 185.0 m a.s.l.). The steepness of the slope was 9–11°. The soil of the southern exposition slope was slightly eroded Eutric Retisol (loamic) (RT-eu.lo), with a texture of sandy loam. The research slope was 65 m in length, and the strip was 3.2 m in width [20].

The agrochemical, physical and biological properties of the soil are presented in Table 1. Changes in the soil’s chemical and physical properties depended on the hill relief. In the downslope direction, humidity and organic carbon resources increased, whereas the soil acidity and the quantity of mobile phosphorus decreased. 

### 2.2. Trial Factors and Treatments

Factor A. Agrophytocenosis: (1) permanent grassland, (2) cereal–grass crop rotation, (3) crop rotation with black fallow. Factor B. Part of the hill: (1) summit, (2) midslope, (3) footslope.

The mixture of perennial grasses for permanent grasslands, which consisted of 20% timothy grass (*Phleum pratense* L.), 20% red fescue (*Festuca rubra* L.), 20% meadow grass (*Poa pratensis* L.), 20% white clover (*Trifolium repens* L.) and 20% common bird’s-foot trefoil (*Lotus corniculatus* L.), was sown in 1993. The grasslands were used without fertilization (abandoned grassland).

The six-course cereal–grass crop rotation consisted of *Hordeum vulgare* L. with undersown perennial grasses (2016), perennial grasses (2017), perennial grasses (2018), *Triticum aestivum* L. (winter crop) (2019), *Hordeum vulgare* (2020) and *Triticum aestivum* (spring crop) (2021).

The six-course crop rotation with black fallow consisted of perennial grasses (2016), *Hordeum vulgare* L. (2017), black fallow (2018), *Triticum aestivum* L. (winter crop) (2019), *Solanum tuberosum* L. (2020) and *Hordeum vulgare* L. with undersown perennial grasses (2021).

For growing the cereal crops, reduced soil cultivation (12–15 cm depth) was applied in the spring. Stubble breaking was performed first, followed by cultivation. During the winter, stubble was left on the slope to secure its soil from erosion.

Before planting the potatoes in the spring, the soil was deep ploughed and cultivated, and during the growing season, the culture of potatoes was disced and hoed one time.

All soils were equally fertilized with granular mineral fertilizers (background fertilization). For fertilizers, N_60_P_60_K_60_ was applied for spring barley as well as for spring wheat, and N_90_P_90_K_90_ was applied for potatoes. Before sowing, the grains of spring wheat and spring barley were treated with Kinto (i.e., triticonazole + prochloraze) at a rate of 2 L t^−1^. Plant protection products (pesticides) were used as well. In 2020, BBCH 32–MCPA (i.e., MCPA 750 g L) was used with 30 mL, and Arrat (i.e., dicam-ba-sodio + tritosulfuron) was used with 10 mL. In 2021, BBCH 33–Trimmer 50 SG (i.e., tribenuron metil) was used with 15 g ha^−1^, and Elegant 2FD (i.e., florasulam 6.25 g L + 2.4-D 300 g L) was used with 0.6 L ha^−1^.

### 2.3. Methods of Analysis

Soil samples were taken from the agrophytocenoses in 2020 and 2021.

To assess the impact of the hill slope on soil contamination by the seeds, the seed bank was investigated at depths of 0–5 and 5–15 cm. The seed bank was analyzed using soil samples taken in the spring (April) and autumn (September) of 2020 and 2021. In each model plot, 2 kg of soil from 20 positions was collected using a hand auger. The soil was dried out. In total, five 100 g samples were removed from 2 kg of soil sample and weighed. Later, the soil samples were wet sieved through a 0.25 mm sieve until all the contents of the soil were washed out. The remaining mineral part of the soil was separated from the organic part and seeds using the saturated salt solution. The seeds were identified using binoculars with 8.75× magnification. Seed viability was determined with “destructive crushing” using forceps [21]. The number of viable seeds (A) was recalculated to thousands of seeds per m^2^.

A = n × h × p × 100,(1)

where A is the number of viable seeds in thousands of seeds per m^2^; n is the counted number of viable seeds in the soil sample; h is the depth of the plough layer in cm; and p is the soil bulk density in g cm^3^.

The Latin names of seed species were presented using the book “Fruits and seeds of Lithuanian plants” [22]. Morphological seed surface and shape traits were described according to Grigas [22]. Different seed shapes (egg-shaped, oval, flattened or round) and surface traits (seeds with a smooth or rough (wrinkled and furrowed) and a shiny or matte surface) were analyzed.

Chemical analyses were carried out at the Chemical Research Laboratory of the Institute of Agriculture, Lithuanian Research Centre for Agriculture and Forestry. Before establishing the experiment, soil agrochemical characteristics were determined from the samples taken at depths of 0–5 and 5–15 cm. Soil acidity (pH) was measured using the potentiometric method with the extraction of 1 M of KCl (pH_KCl_), according to the International standard ISO 10390:2005 (soil quality determination of pH). In the soil, mobile P_2_O_5_ and K_2_O were determined using the Egner–Riehm–Domingo (AL) method (LVP D-07:2016), total nitrogen (N_tot_) content was determined using the Kjeldahl method, and organic carbon (C_org_) content was determined using the Dumas dry combustion method. Soil bulk density was determined with a 100 cm^3^ cylindrical drill using the Kachinsky method. Soil texture was determined with the Fere triangle (FAO recommended method), according to the percentage of sand, silt and clay fractions in the graphical diagram.

The soil microbial biomass carbon (MBC) was determined with the chloroform fumigation and extraction (CFE) method, according to Vance et al. [23] and Brooke [24].

The MBC of the sample was calculated with the following equation [25]:

MBC = CE/0.35,(2)

where MBC is the amount of the dry weight of air-dry soil obtained from the average of three measurements, expressed in mg C; and CE is the difference between organic C extracted from fumigated and non-fumigated samples.

### 2.4. Statistical Analysis

The significance of the differences between the means was determined according to Fisher’s protected Least Significant Difference (LSD) at a 0.05 probability level. The experimental data were subjected to an analysis of variance (ANOVA) [26]. The actual data of the seed bank were transformed as follows:

(Sqr(x + 1))(3)

To assess the influence of the seed surface on the distribution of seed bank species on different parts of the hill, the average values of seeds in different agrophytocenoses were used. The hierarchical clustering analysis was conducted to investigate how seed shape and surface impact the distribution of seeds on different parts of a hilly relief (summit, midslope and footslope) in spring and autumn. The data were converted to a binary matrix, where the absence of a trait was 0, and the presence of a trait was 1. The dendrogram was constructed using the Nei–Li [27] distance and UPGMA methods in the Treecon v.1.3b program [28]. To test the reliability of the dendrogram, a bootstrap analysis with 1000 replications was performed using the bootstrap tool in Treecon software.

## 3. Results

### 3.1. Seed Reserves in the Soil and the Vertical Distribution of the Soil Seed Bank

**Figure 1:**
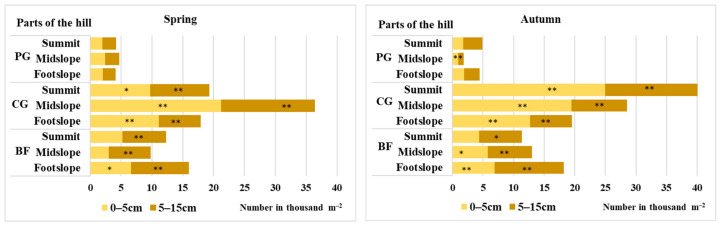
Soil seed bank in different agrophytocenoses, 2020–2021; PG—permanent grassland; CG—cereal–grass crop rotation; BF—crop rotation with black fallow. * and ** indicate significance at *p* ≤ 0.05 and *p* ≤ 0.01, respectively.

The average data of 2020 and 2021 show that, in the spring (the beginning of plant vegetation), the seed number in the soil seed bank of the permanent grassland was determined to be significant and the smallest (4.3 thousand seeds m^−2^) (Figure 1), whereas the highest seed number (24.6 thousand seeds m^−2^) was determined in the soil of the cereal–grass crop rotation. In the soil of perennial grassland, the seed number was 5.6 and 2.9 times smaller compared to those of the cereal–grass crop rotation and crop rotation with black fallow.

The same tendency remained in autumn; the seed number in the soil of perennial grassland was 8.0 and 3.8 times smaller compared to those of the above-mentioned crop rotations. The received differences were significant. Moreover, in autumn, the seed number in the soil of both crop rotations was determined to be 21.2 and 11.7% higher compared to that of spring, respectively, in the cereal–grass crop rotation and in the crop rotation with black fallow. In the permanent grassland soil, in the autumn, the seed number was 14.1% smaller than that in the spring.

Although the hill parts did not have a significant influence on the number of seeds, in the spring at both soil depths of the midslope, the number of seeds (irrespective of the agrophytocenosis) was determined to be 42.2 and 34.0% higher compared to those of the summit and footslope parts of the hill. In autumn, the seed number at both soil depths was 31.1 and 34.7% higher than that of the summit of the hill compared to the midslope and footslope parts.

**Figure 2:**
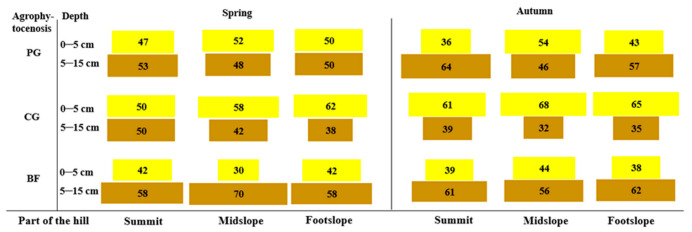
Vertical distribution of soil seed bank (%) in 2020–2021. PG—permanent grassland; CG—cereal–grass crop rotation; BF—crop rotation with black fallow.

The vertical distribution of the seed reserves depended on the applied soil tillage. For the permanent grassland in spring, the seed number was determined to be similar at both soil depths of all parts of the hill (about 50%). In autumn, only the 0–5 cm soil depth for the summit part of the hill was distinguished by a smaller seed number (37.1%) (Figure 2).

In the cereal–grass crop rotation in the spring, the number of seeds at a depth of 0–5 cm increased in the downslope direction from 53.1 to 60.9%. In autumn at a soil depth of 0–5 cm for all parts of the hill, the seed number was higher than that in the spring and composed 60.2–65.3%. The midslope part of the hill was distinguished by a higher seed number. Both in the spring and in autumn, the smallest seed number was determined in the summit of the hill.

In the crop rotation with black fallow in the spring, a higher seed number was determined for a depth of 5–15 cm for all parts of the hill (56.8–69.8%). The same tendency remained in the soil seed bank in autumn.

### 3.2. The Number of Seed Species

During the investigation period, the seeds of 44 plant species were found in the soil seed bank; 27 species belonged to weeds, 16 species belonged to plants from other growth places, and 1 species belonged to trees. The total seed species number in the cereal–grass crop rotation site was 24 (Tables S3 and S4). In the crop rotation with black fallow, it reached 28 (Tables S5 and S6), and in the grassland, it reached 32 (Tables S1 and S2).

The results of the dispersive analysis show that both examined factors were significant for the number of species in the soil seed bank at depths of 0–5 and 5–15 cm. In most examined cases, the highest number of seed species was determined in the soil of the cereal–grass crop rotation. At a soil depth of 0–5 cm, the seeds of 6 to 11 plant species were found (on average, 8 species), whereas in the lower soil layer, the seeds of 3 to 9 plant species were found (on average, 6 species) (Table 2). In all examined cases, the highest significant number of seed species was determined in the footslope of the hill.

### 3.3. Seed Surface Morphological Traits of Soil Seed Bank

**Figure 3:**
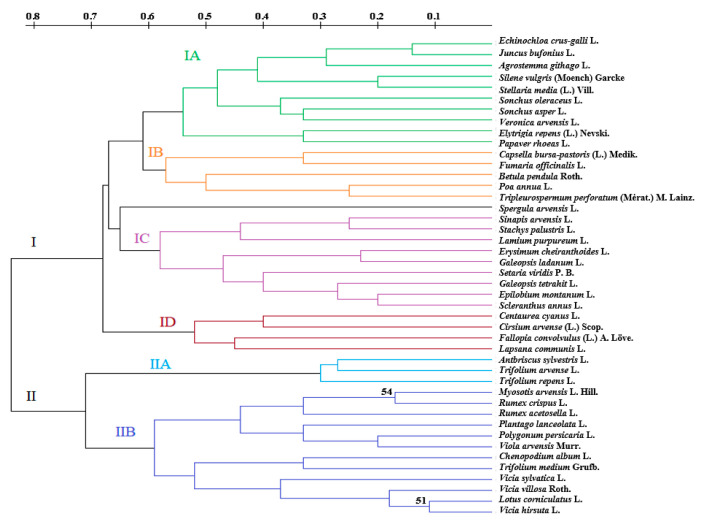
Hierarchical cluster analysis of soil seed bank using 27 morphological traits of seed shapes and surfaces. Roman numerals indicate the main clusters and subclusters according to different features of seeds, such as the surface (I—rough, II—smooth, IA—matte, IB—wrinkled, ID—furrowed, IIA—slightly shiny, IIB—shiny) or shape (IC—egg-shaped). The scale above the dendrogram indicates the distance coefficient between soil seed banks using morphological traits of seeds. Arabic numerals above branches indicates the bootstrap values in percentages.

In the hierarchical cluster analysis, all seeds were separated into two main clusters according to the seed surfaces during spring and autumn (Figure 3 and Figure S1). By analyzing the influence of the seeds’ shape and surface in the soil seed bank on the spread, it was determined that seed surface was a major factor. In the spring, 23 species of seeds with rough surfaces were distinguished in the summit, 19 species were distinguished in the midslope, and 20 species were distinguished in the footslope of the hill. In the autumn, respectively, 19, 17 and 22 species of the seeds with rough surfaces were distinguished in the summit, midslope and footslope parts. The seeds of *Trifolium repens* L. were found in all hill parts in the spring, whereas the seeds of *Silene vulgaris* (Moench) Garcke (Figure S1A–F, respectively) were found in all hill parts in autumn. The seeds of *Stachys palustris* L. (Figure S1A,B) were found only in the summit of the hill in both spring and autumn. In the spring, the seeds of *Lapsana communis* L., *Epilobium montanum* L., *Galeopsis ladanum* L., *Poa annua* L. and *Plantago lanceolata* L. (Figure S1A) were identified only in the summit of the hill. The seeds of *Centaurea cyanus* L. were identified only in the summit of the hill in spring and in the footslope of the hill in autumn (Figure S1A,F), whereas the seeds of *Scleranthus annus* L. were found only in the summit of the hill in autumn (Figure S1B).

The number of seeds with rough surfaces dominated in both spring and autumn compared to the number of seeds with smooth surfaces. In the spring, the number of seed species with rough surfaces was 2.0–3.3 times higher, and in the autumn, it was higher by 2.1–2.8 times.

By estimating the importance of the seeds’ shape and surface of the seed bank on the spread, no significant differences were determined between the hill parts in spring and autumn. A total hierarchical clustering analysis of all hill parts and both seasons was conducted, showing that all seeds with rough surfaces were separated (29 in total) species in the first cluster (I). All seeds with smooth surfaces (14 in total) belonged to the second main cluster (II), with the exception of *Trifolium repens* L. with a rough surface and appearing only in the spring (Figure 3). This shows the dominance of rough-surfaced seeds at all hill sites in spring and autumn, with an average of 57% and 69.6%, respectively. Further division of main clusters into subclusters was determined according to different seed shapes and surfaces. In the first cluster, *Spergula arvensis* L. was separated from other members according to its round seeds and ribbed surface. Another three clusters were separated according to matted (IA), wrinkled (IB) and furrowed (ID) surfaces and egg-shaped seeds (IC, Figure 3). In the second cluster, the first level of the grouping of seeds was determined according to slightly shiny (IIA) or shiny (IIB) surfaces. Further division into subclusters was performed according to the seed shape (egg-shaped, oval, flattened or round).

Key features of the seeds’ surfaces, which separated the soil seed bank into two groups according to the hierarchical cluster analysis, differed among hill sites. In spring and autumn, the highest number of seeds with rough surfaces (63.2 and 75.9%) was determined in the summit of the hill. Going down from the summit to the footslope, the number of seed species with rough surfaces reduced. In the midslope of the hill, the number of seeds with rough surfaces reached 68.6 and 65.4%, respectively, in spring and in autumn, whereas in the footslope of the hill, this number composed 57.9 and 56.2% (in spring and in autumn, respectively). The highest number of seeds with smooth surfaces was determined in the footslope of the hill in spring and autumn, respectively, composing 42.1 and 43.8%.

### 3.4. Correlation between the Seed Number and Soil Microbial Biomass Carbon

By analyzing changes in the soil seed bank, the total correlation between the seed number and soil microbial biomass carbon was estimated. In the spring as well as in autumn, the microbial biomass carbon content basically depended on the hill part (*p* ≤ 0.01). In the summit of the hill, MBC content was determined to be significantly smaller (on the average 319.67 µg C g^−1^) (Table 1). In the midslope and footslope of the hill, the MBC content was 26.6 and 27.6% higher than that in the summit of the hill.

The seed number correlated with the soil microbial carbon biomass. Under hilly relief conditions in the spring, there were no reliable relations determined between these factors. However, in autumn, the dispersive analysis showed that the microbial biomass carbon correlated with the seed number. In the footslope part of the permanent grassland, the correlation between these factors was strong at depths of 5–15 cm and 0–15 cm (r = 0.902, *p* ≤ 0.05 and 0.846, *p* ≤ 0.05). The dependence of soil microbial biomass carbon content on the cereal–grass crop rotation in the midslope of the hill at a depth of 0–15 cm (r = −0.859, *p* ≤ 0.05), as well as the crop rotation with black fallow in the midslope of the hill at depths of 0–5 cm and 0–15 cm (r = 0.841, *p* ≤ 0.05 and 0.922, *p* ≤ 0.01), was also determined.

## 4. Discussion

### 4.1. Seed Reserves in the Soil and the Vertical Distribution of the Soil Seed Bank

Literature sources indicate that soil seed banks are a key to understanding the dynamics of plant populations, species and ecosystems [29]. Different vegetation types vary in their seed bank characteristics, such as seed density and seed bank species richness [30,31].

The small number of seeds in the soil of permanent grassland coincides well with data from research accomplished in Estonia [32]. Data from research conducted in Lithuania show that the soil seed banks of the permanent grasslands were composed of arable weeds; *Chenopodium album* L. was the dominant species in the soil seed bank of the summit of the hill (0–15 cm depth), whereas *Stellaria media* (L.) Vill. dominated the soil seed bank of the midslope, composing 31.3 and 18.4% of the total seed number, respectively [33]. The studied area underwent changes in land use from arable land to grassland. Therefore, in the meadow soils of Spain, over half of seed banks are often composed of arable weeds, creating long-term persistent seed banks that are non-significant for the structure of meadow communities [34]. Seeds accumulated in meadow soils often do not correspond to the species composition of meadow communities in Hungary [35]. The species composition of a soil seed bank corresponded to approximately 30–40% of the botanical composition of meadow swards in Poland [36].

In cereal–grass crop rotation, where reduced soil tillage was applied, the seed number in the soil was significantly the highest. Auškalnienė et al. [12] also reported about the high seed density in the reduced tillage system, as compared to the conventional system. A higher seed number in the soil is related to higher crop weediness applying reduced soil tillage [37,38]. Reduced soil tillage promotes the spread of weeds [13,39].

By growing cumulative plants, the conventional soil tillage system was applied. The number of seeds in the soil was determined to be two times as small compared to cereal–grass crop rotation, where reduced soil tillage was applied. Previous studies have also indicated that the soil seed bank is 1.5 and 2.2 times greater with the shallow ploughing and shallow ploughless tillage treatments compared with that of the conventional tillage treatment [38,40].

In other studies, it was stated that winter cereals, sunflowers and lupines increase the weed seed bank by 30–40%; grass–clover mixtures, however, reduce the seedbank by 39% [41].

The vertical distribution of seed resources depended on the agrophytocenosis. The permanent grassland constantly provided anti-erosion protection for the slope soil (for over 27 years). In this situation, grass vegetation was a physical–biological barrier for water runoff from the slope surface [33]. After a sward is formed, slopes of the hill are protected from running water flows [42]. In the spring (the beginning of plant vegetation), in the soil of all hill parts in the permanent grassland, the seed number at both depths was determined to be similar. In autumn, a depth of 0–5 cm in the summit of the hill was distinguished by a smaller seed number. This could have been influenced by environmental factors (wind and precipitation), which promote the annual spread of new seeds during the plant vegetation period [43,44].

In cereal–grass crop rotation, due to applied reduced soil tillage, the seed number at a depth of 0–5 cm was higher compared to that of a depth of 5–15 cm, and it increased in the downslope direction. This tendency was determined in the seed bank in both spring and autumn [19]. By applying reduced soil tillage, around 80–90% of weed seeds spread in the topsoil layer to a depth of 10 cm. A major part of weed seeds in no-tillage fields remain in the soil surface or close to it [45]. Under conventional tillage systems, recently dispersed seeds of any weed species are found in the top 5 cm only after crop harvest. At this time, older seed generations are in deeper layers of the soil [3].

In the rotation where the cumulative plants were grown, the soil was more cultivated. A higher seed number was determined at a depth of 5–15 cm. The annual ploughing of cereal fields can bury seed surfaces to below germination depth but can bring seeds from previous years up to near the surface [46]. Some studies have shown that intensive soil cultivation, such as moldboard plowing, which turns soil up to 20 cm, has a more uniform distribution of seeds in its soil profile [38,47].

### 4.2. The Number of Seed Species

Different-intensity agrophytocenoses and hill parts had a significant influence on the number of seed species. The total number of species was variable among the examined sites, but in general, it was lower at the crop rotation sites than that at the sites located in grasslands (Table 2). Consequently, the number of plant seed species in the soil seed bank also depended on different management, which was applied to agrophytocenoses. Arable fields differ from natural habitats, as they are more nutrient-rich and highly disturbed environments. In the shallow ploughing and shallow ploughless tillage treatments, 25.5 and 41.5% more weed seed species were found in the soil compared with those of the conventional tillage treatment [38].

### 4.3. Seed Surface Morphological Traits of Soil Seed Bank

Plants are adapted to disperse their seeds in a variety of ways [44,48]. The morphological characteristics of seeds are related not only to the identification of the seed species, but also to the distribution of seeds in nature [49]. Specific morphological features of seeds are some forms of adaptation to seed dispersal [50]. According to the results of this study, seed surface was one of the main factors determining seed dispersal (Figure 3). In the cluster analysis, all species of the soil seed bank were separated into two main groups: species with smooth surfaces in one cluster, and those with rough surfaces in the second. The highest number of seeds with rough surfaces was observed in the summit, irrespective of the season (Figures S1–S6). Going down toward the footslope, the ratio of seeds with rough and smooth surfaces changed. The number of seeds with rough surfaces decreased, and the number of smooth seeds increased. This was likely due to the fact that, in a hilly relief, seeds are easily carried by water currents when snow melts or downpours pass [51]. In addition, seeds with a rough surface are more likely to stick to the soil surface in a hilly relief than those with a smooth surface. Seed shape also influences hydrodynamic behavior via selective entrainment and preferential deposition [46]. Seed shape was a secondary key factor for distribution of the soil seed bank in this study, as the separation of species in subclusters was performed at the second or third level of the dendrogram in the cluster analysis. The main seed shape features for further division into subclusters were egg-shaped, oval, flattened or round. In the cluster analysis, there was no clear separation in the seed soil bank according to the season or different sites of the hill. This could have been caused by relatively small height differences between the summit and footslope (about 10–13 m). Another reason for the absence of separation could have been the fact that the soil seed bank in the spring was persistent, and in autumn, the entire soil seed bank was detected [52]. Therefore, only the ration of seeds differed between spring and autumn.

### 4.4. Correlation between the Seed Number and Soil Microbial Biomass Carbon

The soil seed bank plays an important role in the natural environment of many ecosystems, functioning as natural seed reserves for the future regeneration of many plant species. Seed mortality in the soil is one of the key factors affecting the persistence and density fluctuations of plant populations, especially for annual plants. A number of soil microorganisms are known for their important relationships with plants, including seedling development and giving rise to numerous rhizosphere processes and disease protection. However, the role of microorganisms, as important regulators of seed banks, has not been well studied [1,53]. Select soil fungi have been shown to cause significant decay in weed seeds [54].

The literature indicates that the insertion of residues stimulates microbiological processes in the soil, and part of the weeds mineralizes more rapidly [55,56,57,58]. During the period of sample taking in the spring (the first decade in April), the air temperature was 6.2 °C, and the soil temperature reached only 4.0 °C. Therefore, it was too cold for high microorganism activity, and no correlation was observed. In autumn, a strong significant correlation was determined between MBC and the seed number in the footslope part of permanent grassland. This correlation showed the most favorable conditions for grassland naturalization according to the chemical, physical and biological factors of the soil [59]. Moreover, a strong negative correlation in the cereal–grass crop rotation was determined in the midslope of the hill. Due to crops grown in the cereal–grass crop rotation, the microbial biomass carbon content decreased (Table 1). Therefore, the activity of microorganisms became weaker, and less nitrogen was loosened for plant needs [60]. In the crop rotation with black fallow, a strong positive correlation was determined in the midslope of the hill. It can be assumed that the application of deep ploughing stimulates organic matter mineralization in the top soil. Thus, the microbial biomass carbon content increases, and nitrogen transformation becomes more intensive as well. Plants also access nitrogen faster [61]. Consequently, the soil seed bank is affected positively. Other research also indicates that soil characteristics, such as concentrations of organic carbon and total nitrogen, play an indirect role in arable seedbanks through their effect on parent plant growth, thereby altering the reproductive potential of different species or plant functional types [62,63].

## 5. Conclusions

In hilly landscape ecosystems, pre-erosion cereal–grass crop rotations and perennial grasslands are highly recommended, as they stabilize erosional processes [15]. When protecting hilly fields, it is difficult to apply regular agricultural techniques. Reduced soil tillage application is one of the most important means for soil protection in hill slopes [64,65]. In cereal–grass crop rotation, where reduced soil tillage was applied, the seed number in the soil was significantly the highest. For growing cumulative plants, the conventional soil tillage system was applied. The number of seeds in the soil was determined to be twice as small compared to the cereal–grass crop rotation, where reduced soil tillage was applied. In the soil of perennial grassland, the seed number was 5.6 and 2.9 times smaller compared to the cereal–grass crop rotation and the crop rotation with black fallow.

As the soil was covered with permanent grass, erosional processes and seed transportation could not happen. On average, in all parts of the hill in the soil of permanent grassland, the number of seeds at both depths was determined to be similar. In the cereal–grass crop rotation, due to applied reduced soil tillage, the seed number at a depth of 0–5 cm was higher compared to that at a depth of 5–15 cm, and it increased in the downslope direction. In the rotation where the cumulative plants were grown, the soil was more cultivated. A higher seed number was determined at a depth of 5–15 cm.

The total number of species was lower at the crop rotation sites than those at the sites located in grasslands.

Seeds with rough surfaces dominated in all parts of the hill, but the highest amount (69.6% on average) was determined in the summit of the hill. In autumn, a strong correlation was found between the total seed number and soil microbial carbon biomass (r = 0.841–0.922).

## Figures and Tables

**Table 1:** Agrochemical, physical and biological properties of the arable (0–15 cm) soil depth (2020).

Soil Properties	Part of the Hill					
	Summit		Midslope		Footslope	
	0–5 cm	5–15 cm	0–5 cm	5–15 cm	0–5 cm	5–15 cm
Permanent grassland						
Soil acidity (pH_KCl_)	5.5	5.8	6.4	6.0	5.1	4.9
Mobile P_2_O_5_ (mg/kg)	52.7	39.7	96.0	46.3	38.3	14.0
Mobile K_2_O (mg/kg)	253.7	138.3	250.7	132.3	329.7	154.0
Total N (%)	0.132	0.096	0.150	0.124	0.162	0.101
Organic C (%)	1.12	0.97	1.54	1.17	1.65	1.12
Soil density Mg m^−3^	1.00	1.12	0.76	0.93	0.81	1.07
Soil moisture ^1^ (%)	21.4–25.5	14.5–18.0	22.3–42.7	22.3–23.4	23.0–30.7	19.5–20.7
MBC µg/g C	278.11	252.83	324.67	287.50	333.17	256.17
Cereal–grass crop rotation						
Soil acidity (pH_KCl_)	5.6	5.4	5.3	5.1	5.1	5.1
Mobile P_2_O_5_ (mg/kg)	192	201	165	168	149	148
Mobile K_2_O (mg/kg)	209	112	198	98	223	107
Total N (%)	0.078	0.077	0.097	0.096	0.106	0.101
Organic C (%)	0.9	0.8	1.1	1.0	1.1	1.0
Soil density Mg m^−3^	1.34	1.36	1.35	1.35	1.27	1.33
Soil moisture ^1^ (%)	12.1–16.1	11.9–14.7	15.4–21.2	14.1–19.4	17.8–21.8	16.9–20.6
MBC µg/g C	210.28	196.33	293.11	278.67	354.00	285.56
Crop rotation with black fallow						
Soil acidity (pH_KCl_)	6.4	6.6	5.4	5.7	5.2	5.4
Mobile P_2_O_5_ (mg/kg)	211.7	213.7	174.3	174.0	163.7	140.0
Mobile K_2_O (mg/kg)	181.7	112.7	225.0	103.7	207.3	116.3
Total N (%)	0.072	0.057	0.084	0.082	0.091	0.085
Organic C (%)	0.65	0.68	0.82	0.82	0.94	0.82
Soil density Mg m^−3^	1.32	1.38	1.30	1.30	1.38	1.44
Soil moisture ^1^ (%)	13.0–15.7	13.3–14.6	15.3–16.6	15.4–16.4	18.2–20.2	17.5–18.9
MBC µg/g C	192.28	220.00	335.22	285.17	354.83	325.61

^1^—Min–max. values during the growing season. MBC—microbial biomass carbon.

**Table 2:** Number of seed species in soil, 2020–2021.

Treatment	Spring		Autumn	
	0–5 cm	5–15 cm	0–5 cm	5–15 cm
Agrophytocenosis (factor A)				
Permanent grassland	8.8 a	4.3 b	7.3 b	3.8 b
Cereal–grass crop rotation	8.4 a	6.6 a	8.6 a	6.6 a
Crop rotation with black fallow	7.2 b	6.0 a	7.6 b	6.9 a
Part of the Hill (factor B)				
Summit	7.2 b	5.1 b	6.4 c	4.4 c
Midslope	7.6 b	5.8 a	7.9 b	6.0 b
Footslope	9.6 a	5.9 a	9.2 a	6.8 a
	F (*t*-test)			
Factor A	ns	**	*	**
Factor B	**	ns	**	**
Interaction of factors A × B	ns	ns	ns	*

Letters a–c indicate significant (*p* ≤ 0.05) differences between the means; * and ** indicate significance at *p* ≤ 0.05 and *p* ≤ 0.01, respectively; ns—not significant.

## Data Availability

Not applicable.

## Supplementary Materials

The following supporting information can be downloaded at: https://www.mdpi.com/article/10.3390/plants12051084/s1, Figure S1: Hierarchical cluster analysis of soil seed bank using 27 morphological traits of seed shape and surface; Table S1: Seed species composition of permanent grassland soil (%) in the depth of 0–15 cm in Spring; Table S2: Seed species composition of permanent grassland soil (%) in the depth of 0–15 cm in Autumn; Table S3: Seed species composition of cereal-grass crop rotation soil (%) in the depth of 0–15 cm in Spring; Table S4: Seed species composition of cereal-grass crop rotation soil (%) in the depth of 0–15 cm in Autumn; Table S5: Seed species composition of crop rotation with black fallow soil (%) in the depth of 0–15 cm in Spring; Table S6: Seed species composition of crop rotation with black fallow soil (%) in the depth of 0–15 cm in Autumn.
