# Supplementary material for: Seed Bank Community under Different-Intensity Agrophytocenoses on Hilly Terrain in Lithuania
Source: Plants (Basel). 2023 Mar 1;12(5):1084. doi: 10.3390/plants12051084 (PMC10005566; doi:10.3390/plants12051084)
Supplement: Supplementary file 1 [file plants-12-01084-s001.zip › Figure S1.pdf]

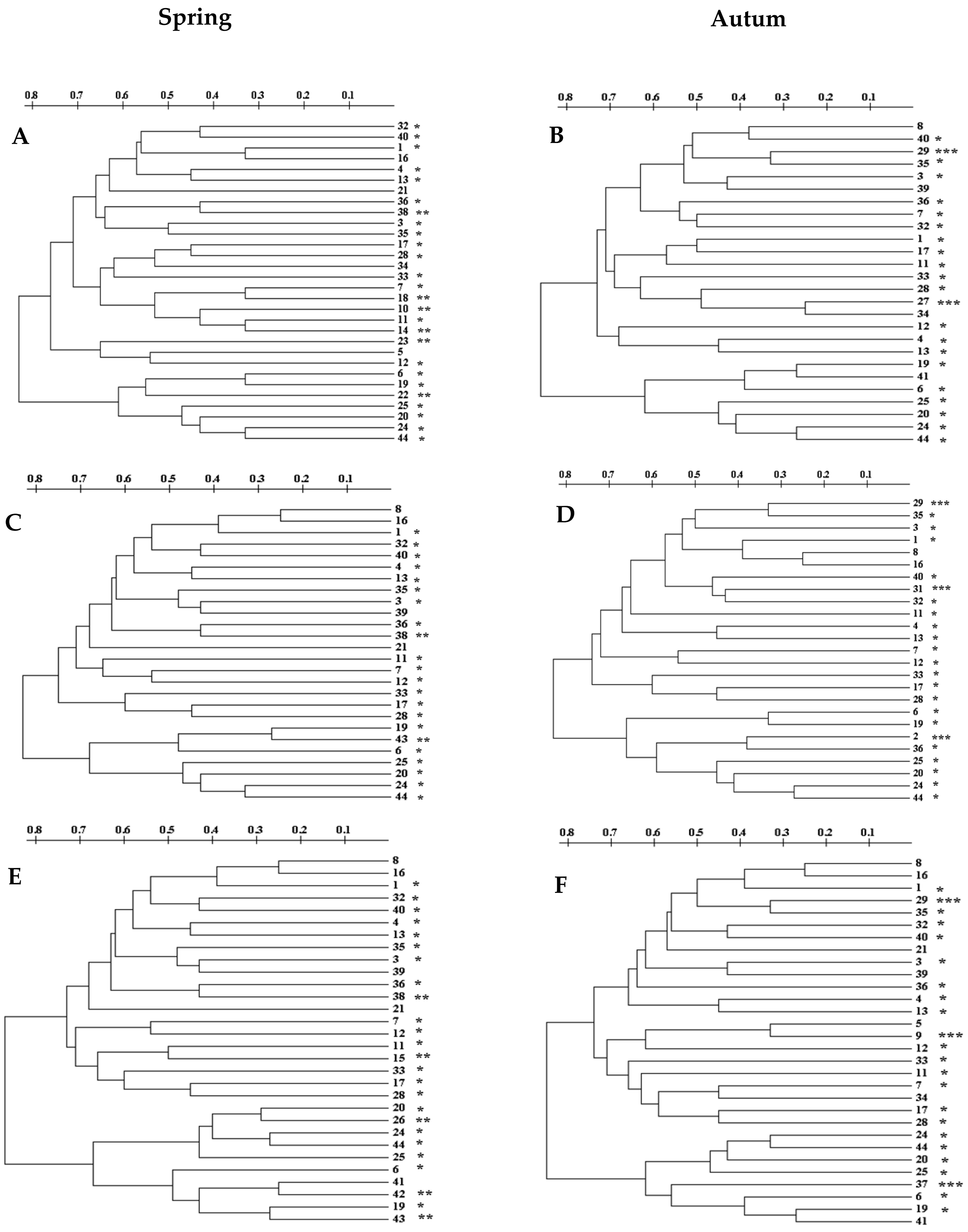
Spring
Autum
0.8 0.7 0.6 0.5 0.4 0.3 0.2 0.1
A
32 *
40 *
1 *
16
4 *
13 *
21
36 *
38 **
3 *
35 *
17 *
28 *
34
33 *
7 *
18 **
10 **
11 *
14 **
23 **
5
12 *
6 *
19 *
22 **
25 *
20 *
24 *
44 *
0.8 0.7 0.6 0.5 0.4 0.3 0.2 0.1
B
8
40 *
29 ***
35 *
3 *
39
36 *
7 *
32 *
1 *
17 *
11 *
33 *
28 *
27 ***
34
12 *
4 *
13 *
19 *
41
6 *
25 *
20 *
24 *
44 *
0.8 0.7 0.6 0.5 0.4 0.3 0.2 0.1
C
8
16
1 *
32 *
40 *
4 *
13 *
35 *
3 *
39
36 *
38 **
21
11 *
7 *
12 *
33 *
17 *
28 *
19 *
43 **
6 *
25 *
20 *
24 *
44 *
0.8 0.7 0.6 0.5 0.4 0.3 0.2 0.1
D
29 ***
35 *
3 *
1 *
8
16
40 *
31 ***
32 *
11 *
4 *
13 *
7 *
12 *
33 *
17 *
28 *
6 *
19 *
2 ***
36 *
25 *
20 *
24 *
44 *
0.8 0.7 0.6 0.5 0.4 0.3 0.2 0.1
E
8
16
1 *
32 *
40 *
4 *
13 *
35 *
3 *
39
36 *
38 **
21
7 *
12 *
11 *
15 **
33 *
17 *
28 *
20 *
26 **
24 *
44 *
25 *
6 *
41
42 **
19 *
43 **
0.8 0.7 0.6 0.5 0.4 0.3 0.2 0.1
F
8
16
1 *
29 ***
35 *
32 *
40 *
21
3 *
39
36 *
4 *
13 *
5
9 ***
12 *
33 *
11 *
7 *
34
17 *
28 *
24 *
44 *
20 *
25 *
37 ***
6 *
19 *
41

Figure S1. Hierarchical cluster analysis of soil seed bank using 27 morphological traits of seed shape and surface. **A** and **B** summit of the hill; **C** and **D** – midslope of the hill; **E** and **F** – footslope of the hill. * – seed species, determined in spring and autumn in all parts of the hill; ** – seed species, determined in spring in all parts of the hill; *** – seed species, determined in autumn in all parts of the hill. The scale above the dendrogram indicates the distance coefficient between soil seeded bank using morphological traits of seeds. Arabic numerals indicate the seed species: 1 – *Agrostemma githago* L.; 2 – *Antbriscus sylvestris* L.; 3 – *Betula pendula* Roth.; 4 – *Capsella bursa-pastoris* (L.) Medik.; 5 – *Centaurea cyanus* L.; 6– *Chenopodium album* L.; 7 – *Cirsium arvense* (L.) Scop.; 8 – *Echinochloa crus-galli* L.; 9 – *Elytrigia repens* (L.) Nevski. ; 10 – *Epilobium montanum* L.; 11 – *Erysimum cheiranthoides* L.; 12 – *Fallopia convolvulus* (L.) A. Löve.; 13 – *Fumaria officinalis* L.; 14 – *Galeopsis ladanum* L.; 15 – *Galeopsis tetrahit* L.; 16– *Juncus bufonius* L.; 17 – *Lamium purpureum* L.; 18 – *Lapsana communis* L.; 19 – *Lotus corniculatus* L.; 20 – *Myosotis arvensis* L. Hill.; 21 – *Papaver rhoeas* L.; 22 – *Plantago lanceolata* L.; 23 – *Poa annua* L.; 24 – *Polygonum persicaria* L.; 25 – *Rumex acetosella* L.; 26 – *Rumex crispus* L.; 27 – *Scleranthus annus* L.; 28 – *Setaria viridis* P. B.; 29 – *Silene vulgaris* (Moench) Garcke; 30 – *Sinapis arvensis* L.; 31 – *Sonchus asper* L.; 32 – *Sonchus oleraceus* L.; 33 – *Spergula arvensis* L.; 34 – *Stachys palustris* L.; 35 – *Stellaria media* (L.) Vill.; 36 – *Trifolium arvense* L.; 37 – *Trifolium medium* Grufb.; 38 – *Trifolium repens* L.; 39 – *Tripleurospermum perforatum* (Mėrat.) M. Lainz.; 40 – *Veronica arvensis* L.; 41 – *Vicia hirsuta* L.; 42 – *Vicia sylvatica* L.; 43 – *Vicia villosa* Roth.; 44 – *Viola arvensis* Murr.
