# Supplementary material for: Seed Bank Community under Different-Intensity Agrophytocenoses on Hilly Terrain in Lithuania
Source: Plants (Basel). 2023 Mar 1;12(5):1084. doi: 10.3390/plants12051084 (PMC10005566; doi:10.3390/plants12051084)
Supplement: Supplementary file 1 [file plants-12-01084-s001.zip › Table S1-6.pdf]

plants

MDPI

Table S1. Seed species composition of permanent grassland soil (%) in the depth of 0–15 cm in Spring.

Plant species	Family	Parts of the hill					
		Summit		Midslope		Footslope	
		2020	2021	2020	2021	2020	2021
Chenopodium album L.	*Amaranthaceae*	33.8	28.7	14.1	12.6	8.0	21.4
Centaurea cyanus L.	*Asteraceae*		6.9				
Cirsium arvense (L.) Scop.	*Asteraceae*	7.7	4.0	9.1	9.2	2.7	14.3
Lapsana communis L.	*Asteraceae*		1.0				
Sonchus oleraceus L.	*Asteraceae*	3.1	1.0	1.0	1.7	3.5	4.5
Betula pendula Roth.	*Betulaceae*	3.1	1.0				
Myosotis arvensis L.	*Boraginaceae*		1.0				
Capsella bursa-pastoris (L.) Medik.	*Brassicaceae*	1.5					
Erysimum cheiranthoides L.	*Brassicaceae*		7.9		0.6		2.7
Stellaria media (L.) Vill.	*Caryophyllaceae*	12.3	9.9	24.2	12.6	2.7	3.6
Silene vulgaris L.	*Caryophyllaceae*	12.3		20.2	33.3	8.0	2.7
Spergula arvensis L.	*Caryophyllaceae*	3.1	1.0		1.7	0.9	
Lotus corniculatus L.	*Fabaceae*	1.5	15.8	6.1	19.0	2.7	25.0
Trifolium arvense L.	*Fabaceae*		1.0	3.0		1.8	
Trifolium repens L.	*Fabaceae*		3.0	1.0	2.9		1.8
Vicia cracca L.	*Fabaceae*					1.8	
Vicia hirsuta L.	*Fabaceae*						15.2
Vicia villosa Roth.	*Fabaceae*			1.0			
Juncus bufonius L.	*Juncaceae*		2.0		1.1		0.9
Galeopsis ladanum L.	*Lamiaceae*		5.0				
Galeopsis tetrahit L.	*Lamiaceae*					0.9	
Lamium purpureum L.	*Lamiaceae*					1.8	
Stachys palustris L.	*Lamiaceae*	1.5	1.0				
Epilobium montanum L.	*Onagraceae*		4.0				
Fumaria officinalis L.	*Papaveraceae*	10.8		4.0		24.8	
Papaver rhoeas L.	*Papaveraceae*					29.2	0.9
Poa annua L.	*Poaceae*		1.0				
Fallopia convolvulus (L.) A. Löve.	*Polygonaceae*	4.6	1.0	2.0		2.7	
Rumex acetosella L.	*Polygonaceae*		2.0		0.6		
Rumex crispus L.	*Polygonaceae*					0.9	
Veronica arvensis L.	*Scrophulariaceae*	1.5	2.0	2.0	3.5	1.8	
Viola arvensis Murr.	*Violaceae*	3.1		12.1	1.1	6.2	7.1
The number of species		14	21	13	13	17	12

Table S2. Seed species composition of permanent grassland soil (%) in the depth of 0–15 cm in Autumn.

Plant species	Family	Parts of the hill					
		Summit		Midslope		Footslope	
		2020	2021	2020	2021	2020	2021
Chenopodium album L.	*Amaranthaceae*	44.1	72.5	16.1	32.6	16.4	18.1
Anthriscus sylvestris L.	*Apiaceae*				3.2		
Cirsium arvense (L.) Scop.	*Asteraceae*	16.9	3.6	19.6	19.0	7.5	3.1
Sonchus asper L.	*Asteraceae*				1.1		
Sonchus oleraceus L.	*Asteraceae*	5.1	1.2	1.8	7.4	1.5	10.2
Betula pendula Roth.	*Betulaceae*	3.4	1.8	1.8	1.1		
Myosotis arvensis L.	*Boraginaceae*				1.1		
Erysimum cheiranthoides L.	*Brassicaceae*	1.7		1.8			0.8
Agrostemma githago L.	*Caryophyllaceae*	1.7		1.8		3.0	
Stellaria media (L.) Vill.	*Caryophyllaceae*	8.5	1.8	7.1	3.2	1.5	5.5
Silene vulgaris L.	*Caryophyllaceae*		7.8		9.5		3.1
Lotus corniculatus L.	*Fabaceae*		4.2		9.5		33.9
Trifolium arvense L.	*Fabaceae*	3.4		10.7	3.2	6.0	4.7
Trifolium medium Grufb.	*Fabaceae*						1.6
Vicia hirsuta L.	*Fabaceae*		2.4			10.4	7.1
Juncus bufonius L.	*Juncaceae*			12.5			0.8
Lamium purpureum L.	*Lamiaceae*	3.4				1.5	
Stachys palustris L.	*Lamiaceae*	1.7					
Fumaria officinalis L.	*Papaveraceae*	3.4		14.3		40.3	
Papaver rhoeas L.	*Papaveraceae*						0.8
Agropyron repens L.	*Poaceae*						2.4
Echinochloa crus-galli L.	*Poaceae*						1.6
Fallopia convolvulus (L.) A. Löve.	*Polygonaceae*		1.8			1.5	2.4
Polygonum persicaria L.	*Polygonaceae*					1.5	
Rumex acetosella L.	*Polygonaceae*	1.7		1.8			
Veronica arvensis L.	*Scrophulariaceae*	3.4	0.6	10.7	1.1	3.0	
Viola arvensis Murr.	*Violaceae*	1.7	2.4		8.4	6.0	3.9
The number of species		14	11	12	13	13	16

Table S3. Seed species composition of cereal-grass crop rotation soil (%) in the depth of 0–15 cm in Spring.

Plant species	Family	Parts of the hill					
		Summit		Midslope		Footslope	
		2020	2021	2020	2021	2020	2021
Chenopodium album L.	*Amaranthaceae*	23.8	6.6	33.3	2.5	20.3	4.0
Centaurea cyanus L.	*Asteraceae*	1.2					
Cirsium arvense (L.) Scop.	*Asteraceae*		0.2			0.5	
Sonchus oleraceus L.	*Asteraceae*					0.5	
Tripleurospermum perforatum M. Lainz	*Asteraceae*					4.5	0.2
Betula pendula Roth.	*Betulaceae*	4.8	0.2	1.0			0.2
Myosotis arvensis L.	*Boraginaceae*			1.0	0.4	6.4	
Capsella bursa-pastoris (L.) Medik.	*Brassicaceae*		0.2		0.3	1.0	0.7
Erysimum cheiranthoides L.	*Brassicaceae*		1.5	1.5	60.0	6.4	32.1
Stellaria media (L.) Vill.	*Caryophyllaceae*	7.1		1.0		1.5	0.2
Spergula arvensis L.	*Caryophyllaceae*	1.2	8.8		3.7		3.3
Trifolium repens L.	*Fabaceae*					1.0	
Vicia villosa Roth.	*Fabaceae*						0.2
Juncus bufonius L.	*Juncaceae*	1.2		0.5			
Lamium purpureum L.	*Lamiaceae*					1.0	0.9
Fumaria officinalis L.	*Papaveraceae*			0.5		1.5	
Plantago lanceolata L.	*Plantaginaceae*		0.2				
Echinochloa crus-gali L.	*Poaceae*				0.1		1.2
Setaria viridis (L.) P. Beauv.	*Poaceae*	13.1	58.8	1.0	6.4	1.5	12.5
Fallopia convolvulus (L.) A. Löve.	*Polygonaceae*	3.6	3.5	13.8	3.4	13.9	8.3
Polygonum persicaria L.	*Polygonaceae*	1.2	0.5		2.8		7.3
Rumex acetosella L.	*Polygonaceae*		0.7	1.5		1.0	
Veronica arvensis L.	*Scrophulariaceae*	2.4	2.4	3.1	11.3	32.2	12.5
Viola arvensis Murr.	*Violaceae*	40.5	16.5	41.5	9.1	6.9	16.3
The number of species		11	13	12	11	16	15

Table S4. Seed species composition of cereal-grass crop rotation soil (%) in the depth of 0–15 cm in Autumn.

Plant species	Family	Parts of the hill					
		Summit		Midslope		Footslope	
		2020	2021	2020	2021	2020	2021
Chenopodium album L.	*Amaranthaceae*	2.5	6.8	2.6	10.8	4.1	7.6
Cirsium arvense (L.) Scop.	*Asteraceae*					0.4	
Sonchus oleraceus L.	*Asteraceae*				0.3	0.4	0.4
Tripleurospermum perforatum M. Lainz	*Asteraceae*	0.2				0.6	
Betula pendula Roth.	*Betulaceae*	0.1	0.7	0.1		0.6	
Myosotis arvensis L.	*Boraginaceae*	0.1		0.9	0.3	0.2	1.4
Capsella bursa-pastoris (L.) Medik.	*Brassicaceae*	0.1	0.3	0.1			0.7
Erysimum cheiranthoides L.	*Brassicaceae*	0.2		40.0	17.3	20.9	7.6
Stellaria media (L.) Vill.	*Caryophyllaceae*				0.6		0.7
Spergula arvensis L.	*Caryophyllaceae*	4.7	22.1	7.4	11.1	5.3	10.4
Lamium purpureum L.	*Lamiaceae*					1.2	1.8
Fumaria officinalis L.	*Papaveraceae*	0.1					
Echinochloa crus-gali L.	*Poaceae*		55.2		22.8		19.1
Setaria viridis (L.) P. Beauv.	*Poaceae*	79.8	6.6	5.8	4.7	14.8	3.2
Fallopia convolvulus (L.) A. Löve.	*Polygonaceae*	0.8	0.2	0.6	1.8	2.5	12.9
Polygonum persicaria L.	*Polygonaceae*			1.3	2.6	0.8	4.7
Rumex acetosella L.	*Polygonaceae*		0.2	0.2	0.3		
Veronica arvensis L.	*Scrophulariaceae*	0.4	0.2	30.0	3.2	20.9	2.9
Viola arvensis Murr.	*Violaceae*	10.9	7.8	11.0	24.3	27.1	26.6
The number of species		12	10	12	13	14	14

Table S5. Seed species composition of crop rotation with black fallow soil (%) in the depth of 0–15 cm in Spring.

Plant species	Family	Parts of the hill					
		Summit		Midslope		Footslope	
		2020	2021	2020	2021	2020	2021
Chenopodium album L.	*Amaranthaceae*	11.7	31.9	7.4	34.1	5.9	21.3
Cirsium arvense (L.) Scop.	*Asteraceae*	0.4	0.7				0.4
Sonchus oleraceus L.	*Asteraceae*		2.1				0.9
Tripleurospermum perforatum M. Lainz	*Asteraceae*			1.2		7.5	
Betula pendula Roth.	*Betulaceae*				0.8	1.7	
Myosotis arvensis L.	*Boraginaceae*			1.2	2.3	0.8	2.7
Capsella bursa-pastoris (L.) Medik.	*Brassicaceae*	0.8				0.8	0.4
Erysimum cheiranthoides L.	*Brassicaceae*	2.9		2.5	0.8	8.8	4.9
Stellaria media (L.) Vill.	*Caryophyllaceae*	0.4		2.5		0.8	
Spergula arvensis L.	*Caryophyllaceae*	26.8		30.9		24.3	0.4
Vicia hirsuta L.	*Fabaceae*						0.4
Lamium purpureum L.	*Lamiaceae*	0.4		1.9		2.9	
Stachys palustris L.	*Lamiaceae*	0.4					
Fumaria officinalis L.	*Papaveraceae*				0.8		
Papaver rhoeas L.	*Papaveraceae*		0.7		0.8		
Setaria viridis (L.) P. Beauv.	*Poaceae*	17.2	30.6	3.7	7.6		5.8
Fallopia convolvulus (L.) A. Löve.	*Polygonaceae*	2.1	18.1	1.9	24.2	9.2	33.3
Polygonum persicaria L.	*Polygonaceae*	0.4		7.4	3.8	4.2	5.8
Rumex acetosella L.	*Polygonaceae*			0.6	0.8	0.4	
Veronica arvensis L.	*Scrophulariaceae*	6.3	16.0	7.4	0.8	3.8	0.9
Viola arvensis Murr.	*Violaceae*	30.1		31.5	23.5	28.9	22.7
The number of species		13	7	13	12	14	13

Table S6. Seed species composition of crop rotation with black fallow soil (%) in the depth of 0–15 cm in Autumn.

Plant species	Family	Parts of the hill					
		Summit		Midslope		Footslope	
		2020	2021	2020	2021	2020	2021
Chenopodium album L.	*Amaranthaceae*	36.4	16.1	49.2	21.7	31.0	18.4
Centaurea cyanus L.	*Asteraceae*					1.1	
Cirsium arvense (L.) Scop.	*Asteraceae*			1.6	0.4	0.5	0.3
Sonchus oleraceus L.	*Asteraceae*	1.5	0.5		2.5		1.7
Tripleurospermum perforatum M. Lainz	*Asteraceae*					0.5	7.5
Betula pendula Roth.	*Betulaceae*			1.1	0.4		0.3
Myosotis arvensis L.	*Boraginaceae*	1.5		1.6	0.8	5.3	3.9
Capsella bursa-pastoris (L.) Medik.	*Brassicaceae*	1.5				1.1	1.1
Sinapis arvensis L.	*Brassicaceae*				0.4		
Erysimum cheiranthoides L.	*Brassicaceae*			1.1		1.6	6.7
Agrostemma githago L.	*Caryophyllaceae*	0.8		1.1			
Sckeranthus annuus L.	*Caryophyllaceae*	0.8					
Stellaria media (L.) Vill.	*Caryophyllaceae*		0.9			0.5	0.3
Spergula arvensis L.	*Caryophyllaceae*		0.9	0.5			
Lotus corniculatus L.	*Fabaceae*				0.4		
Trifolium arvense L.	*Fabaceae*				0.4		
Lamium purpureum L.	*Lamiaceae*				0.8	2.1	1.4
Stachys palustris L.	*Lamiaceae*						0.3
Fumaria officinalis L.	*Papaveraceae*			1.1			0.3
Echinochloa crus-gali L.	*Poaceae*		35.9		11.5		8.4
Setaria viridis (L.) P. Beauv.	*Poaceae*	37.9	15.2	8.6	3.3	2.7	1.4
Fallopia convolvulus (L.) A. Löve.	*Polygonaceae*	1.5	22.1	4.3	27.5	12.8	26.3
Polygonum persicaria L.	*Polygonaceae*	0.8	2.3	1.6	9.4		3.9
Rumex acetosella L.	*Polygonaceae*		0.5	2.1			0.3
Veronica arvensis L.	*Scrophulariaceae*	0.8	0.5	25.7	0.8	7.5	0.8
Viola arvensis Murr.	*Violaceae*	16.7	5.1	0.5	19.7	33.2	16.8
The number of species		11	11	14	15	13	19
